# An Empirical Investigation of the Continued Usage Intention of Digital Wallets: The Moderating Role of Perceived Technological Innovativeness

**DOI:** 10.1186/s43093-022-00158-0

**Published:** 2022-10-01

**Authors:** Sabakun Naher Shetu, Md. Muzahidul Islam, Sadia Islam Promi

**Affiliations:** 1 Department of Marketing, Faculty of Business Studies, Jahangirnagar University, Savar, Dhaka, 1342 Bangladesh; 2 Department of Business Administration in Marketing, Faculty of Business Studies, Bangladesh University of Professionals, Mirpur Cantonment, Dhaka, 1216 Bangladesh

**Keywords:** Digital wallets, Continued usage intention, Perceived technological innovativeness

## Abstract

The paper examines the constructs that instigate the users to adopt digital wallets and continued usage behavior in a developing country perspective, Bangladesh. The researcher used a cross-sectional design to collect the primary data using a self-administered questionnaire. The population consisted of the youth, precisely 18–35, who are tech-savvy and knowledgeable about new technology. The study followed the nonprobability purposive sampling technique, and 330 responses were collected through a structured questionnaire survey. In direct path analysis, the findings revealed that perceived usefulness, ease of use, compatibility, insecurity, and behavioral intention to adopt digital wallets found significant relationships among the constructs that supported the proposed hypotheses. Moreover, indirect path analysis of perceived compatibility, perceived personal innovativeness and perceived social influence found no significant relationships that did not justify the proposed hypotheses. Users' behavioral intention partially mediates among perceived usefulness, ease of use, personal innovativeness, and perceived social influence, except perceived compatibility. The findings suggested that perceived technological innovativeness did not support the proposed hypothesis. The incorporated constructs of this study have hardly been found in the existing literature, and the researchers shed light on the unexplored research area. The study results, implications, and limitations have been discussed.

## Introduction

Digital wallets and many other mobile payment systems have seen significant growth. Payment techniques for goods and services have changed dramatically in recent decades, increasing global demand for digital and cashless transactions [66]. This change in payment systems is made possible due to several factors such as changes in the economy, the development of the internet, and the availability of mobile devices [18]. At the same time, it is noticed that users favor fast, convenient, and valuable technologies [89]. Factors like standardization, interconnectivity and comprehensive acceptance procedures are essential for rapid digital payment acceptance [100]. As this technology becomes more sophisticated, new payment types have emerged, such as near-field communication (NFC) [66]. Mobile operators and banks collaborate to provide this service [66]. Previously, the mobile telecommunications and financial industries have been divided into discrete sectors and markets [95]. While electronic or online payment systems have seen tremendous growth, according to Tecnocom, mobile payments have not matched initial expectations [4]. Privacy concerns, the complex nature of the system [33], lack of confidence from the users [94], as well as insufficient knowledge [48] regarding the system possess a great challenge for the adoption of the payment system. Despite this early reluctance, it is believed that these payment methods will eventually become mainstream with the advances in mobile technology and the availability of financial services apps [18]. Mobile payment is the fastest-growing application.

An Indonesian study found that education and income level were positively associated with perceived readiness to go cashless found by an Indonesian survey [7]. Factors like perceived usefulness, performance expectations, ease of use, and perceived compatibility contribute significantly to mobile payment adoption [63]. Davis [16] developed the Technology Acceptance Model (TAM). The researcher suggested that perceived usefulness and ease of use were the individual factors that determined the attitude toward adopting specific technology. Besides TAM, another popular model, Unified Theory of Acceptance and Use Technology (UTAUT), measures the individual intention to use or adopt digital payment services [86]. The technological adoption in everyday life of the people of Bangladesh has contributed to enormous progress in digital payment services that help to contribute to the cashless economy [34]. People are more willing than in previous years to undertake digital payment services, including mobile payment, synonymously addressed as digital wallets. From Bangladesh's perspective, few types of research have focused on the integrated model of the Technology Acceptance Model (TAM), Diffusion of Innovation (DOI), and Technology Readiness Index (TRI2.0) in terms of digital wallets and the continued usage evaluation.

Therefore, the present study aims to fill these gaps by developing a model to understand the forces influencing the continued use of digital wallets. The study attempts to examine the behavioral factors that drive the users’ toward the continued usage behavior of digital wallets through an integrated framework of the Technology Acceptance Model (TAM) [16], Diffusion of Innovation (DOI) [71], Technology Readiness Index 2.0 (TRI 2.0) [64]. The literature review was constructed to provide further insights into how these elements can contribute to the development of continued usage of digital wallets. The remaining parts are developed as follows: “Literature review” section provides a literature review along with the development of hypotheses, followed by the methodological aspect, which in turn is followed by the data analysis section. Finally, “Conclusions” section report concludes with the findings' implications, limitations, and future direction for further studies.

## Literature review

To begin with, digital wallet payment services are services in which a mobile device is used to settle payments for goods, services, and bills through wireless and other communication technologies [93]. Another prime factor has been observed while ensuring the continued usage, i.e., perceived technological innovativeness. The present study identified the significance of perceived technological innovativeness and adapted it to this current study. However, the issue of relying on digital wallets entirely and eliminating their substitutes still has a long way to go. The term 'innovativeness' is mandatory when analyzing why a factor is prone to accept or would be used effectively while dealing with technological matters. This is why TRI, or Technology Readiness Index, came into account [64]. Several studies have examined how digital wallets have been adopted fruitfully and their future, frequently assessing technological and behavioral factors [38]. The article considers necessary variables like perceived ease of use (PEOU), perceived usefulness (PU), trust, compatibility, cost, norm, payment habit, mobile phone skills availability, and ease of access.

Understanding human behavior and acceptance is essential in this regard—merging the Theory of Reasoned Action (TRA) developed [3] and the Theory of Planned Behavior (TPB) [2]. These theories have been famously practiced as powerful frameworks for understanding the adaptiveness of several IT systems, including that digital wallets. Nevertheless, digital wallets have been scrutinized due to their enormous precariousness [49, 92]. Despite the more excellent rates of online payment being accepted worldwide, the usage of digital wallets seems to carry greater risk because of their vulnerability to being digitally hacked or the information being mistreated or spread while tracking the encrypted data illegally [92]. The prime reason digital wallets cannot be safer is that encryption systems lack vigor and strengthen [101]. A study in Taiwan showed an adverse relationship portraying the increased risks during digital wallets' association with everyday living and showcasing how the users were more inclined to switch to the substitutes of the digital wallets [66].

Digital wallet acceptance has been termed a 'puzzle of abundance' [76]. A study in South Korea drafted the importance of several factors in technology acceptancy, including personal innovativeness, knowledge about mobile payment, accessibility, and convenience, which are proportional to perceived ease of use. Concerning digital economy platforms, companies gain more market power, which entails ensuring their customers' welfare and generating trust through perceived credibility and benevolence [19], and found that a significant positive relationship between confidence and satisfaction is highly ethically responsible digital platforms. As the importance of digital wallets persists, it brings forth a ray of hope to the digital economy platforms and increases market size; the companies focus majorly on creating long-term dependability and benignity [19, 93]. Companies have been increasingly shifting their focus onto ensuring that more services are available for the general public after getting information from the mass audience about the increased acceptance of digital wallets, especially mobile payments [48]. Mobility has been termed a prime factor in ensuring the success of digital wallets, among other means of payment [6]. However, there can be several factors like the extent to which the network is being covered, bandwidth, battery life or duration of the batteries, or the availability of operators offering these services that could eventually reduce the perceived mobility in this case [47].

## Theoretical framework and hypotheses development

The study attempts to examine the behavioral factors that drive the users' toward the continued usage behavior of digital wallets through an integrated framework of the Technology Acceptance Model (TAM) [16], Diffusion of Innovation (DOI) [71], Technology Readiness Index 2.0 (TRI 2.0) [64]. The Technology Acceptance Model (TAM) was developed by Davis [16]. The researcher suggested that perceived usefulness and ease of use were the individual factors that determined the attitude toward adopting specific technology. Consequently, adopting the technology also decides the intention of the individuals [17]. The researchers still consider TAM the most influential and rigorous model related to technology acceptance [16, 17]. Moreover, TAM has been considered a vital model for exploring an individual's intention toward accepting or rejecting new technologies [55]. This model has been applied in different fields of research, such as mobile services [90], mobile wireless [41], mobile ticketing [83], and mobile payment services and systems adaptation [11, 18, 23, 25, 46, 47, 60].

Besides TAM, another popular model, Unified Theory of Acceptance and Use Technology (UTAUT), is considered to measure the individual intention to use or adopt digital payment services [86]. Perceived ease of use, usefulness, facilitating conditions, and subjective norms have consisted of the UTAUT model. Still, Venkatesh et al. [87] extended the UTAUT2 model by adopting additional constructs of innovativeness, perceived risk, attitude, and social influences. The evidence suggests that UTAUT and UTAUT2 constructs' perceived ease of use, perceived usefulness, innovativeness, and social influence have a significant influence on the adoption of e-wallets in south-Asian countries like India and Pakistan [57, 68, 74, 79–81]. In this research model, diffusion of innovation theory [71] contributes by examining innovations which are considered the vital element [99], and users' innovativeness in technology adoption is indicated as the significant outcome of innovation theory [59]. From Bangladesh's perspective, few researchers have focused on the integrated model of TAM, DOI, and TRI2.0 in digital wallets and the continued usage evaluation.

### Perceived usefulness

Lu et al. [51] included the Technology Acceptance Model (TAM) developed [16] to explain how perceived usefulness and ease of use are significant factors that correlate to behavioral intention. Likewise, TAM is a model based upon the Theory of Reasoned Action (TRA) modeled by Ajzen and Fishbein [3]. Perceived usefulness refers to when a technology item seems beneficial to someone to get their desired outcome [88]. On the other hand, if technology is perceived as not applicable, which depends from user to user, no matter how many implementation efforts it persists, it will not be accepted across all boundaries, as portrayed by Kustono in this research among college students [45]. Mun et al. [58] showed how perceived usefulness was the most vital factor affecting consumers' behavioral intention to use TAM digital wallets. Perceived ease of use, usefulness, perceived risk, and attitude significantly affected the intention to use an e-wallet [44]. Based on an extended Expectation Confirmation Model (ECM) [36], the impact of a user's expectation and confirmation on their satisfaction and perceived usefulness is portrayed, which ultimately encourages their behavioral intention to enhance the usage of any new technology. A system termed "usability perception" determines the degree of perceived usefulness to make users agree on the correlation between continued usage and a positive relationship [76].

#### **H1a**

Perceived usefulness positively influences behavioral intention to adopt digital wallets.

#### **H1b**

Users’ behavioral intention to adopt digital wallets mediates the relationship between perceived usefulness and continued usage behavior of digital wallets.

### Perceived ease of use

Ease of use is the term that describes how an individual portrays any process or system to be completed within a short period without much hassle and thus being easy to handle [84]. Henceforth, ease of use is one of the essential variables to consider while researching a consumer's willingness to use. The relationship between ease of use, attitude, and intention to use has also often been examined [75]. Perceived ease of use in digital wallets includes ease of handling, fast processing of the payment transaction, the high number of accepting merchants, easy learnability of payment procedure, no installation of software on the mobile device, and no pre-registration necessary [9]. The era of globalization has primarily converted consumers' digital wallets into everyday activities based on their ease of use [1]. Many researchers have previously studied digital wallets’ convenience among consumers and have demonstrated how perceived ease of use coerces users to continue usage of digital wallets [5, 18, 52, 85, 96]. Studies showed how perceived ease of use could enhance long-term satisfaction among consumers [81]. The Payment and Clearing Association of China study revealed how 95.6% of consumers used mobile payment services based on ease of use and convenience [10]. Keramati et al. [38] focused on how digital wallet services are adopted through a conceptual model where the behavioral intention is variable, including ease of use. Therefore, digital wallet providers should effectively understand how intention and loyalty to continue their usage are positively affected by ease of use or convenience.

#### **H2a**

Perceived ease of use positively influences behavioral intention to adopt digital wallets.

#### **H2b**

Users’ behavioral intention to adopt digital wallets mediates the relationship between perceived ease of use and continued usage behavior of digital wallets.

### Perceived compatibility

A consumer's compatibility refers to the extent to which digital wallets will complement their lifestyles [8]. Therefore, the more compatible the mobile apps seem, the more positive word of mouth (WOM) will be spread among loyal consumers. Lifestyle compatibility explains how an individual behaves and chooses which product or service to use daily [29, 91]. In a similar study, it was mentioned that compatibility with a digital product is related to the previous experiences that the consumer has had with the product [37]. The more excellent mobile payment compatibility with the individual's values, needs, and experiences, the more willing that individual is to try out this technology. The more the individual's social environment favors the new technology, the greater the individual's intention to adopt mobile payment [76]. Today, many consumers have been so compatible with mobile phone apps (also for digital payments) that they have intertwined their lives entirely with them, where their dependency levels have been high [8]. Lifestyle compatibility depends on an individual's previous experience using digital wallets [32]. Based on the purpose of the e-wallet, compatibility is, therefore, one variable that will influence the usefulness of an innovation [70].

#### **H3a**

Perceived compatibility positively influences behavioral intention to adopt digital wallets.

#### **H3b**

Users’ behavioral intention to adopt digital wallets mediates the relationship between perceived compatibility and continued usage behavior of digital wallets.

### Perceived personal innovativeness

From a customer's perspective, personal innovativeness refers to how a customer drives toward discontinuity of a product or when they decide to change or adapt to innovation or a substitute [78]. Therefore, Mancha and Shankaranarayanan [53] investigated digital innovativeness in a platform context, concluding that some online businesses, such as Uber, can be considered "digital innovators" in their successful digital business models, digital wallets will do the same. People interested in new users of the latest technology are generally accustomed to taking high risks and will adopt digital wallets regardless of these risks [85]. Previous empirical findings indicate that perceived personal innovativeness positively affects the intention to use mobile payment services [7, 66].

#### **H4a**

Perceived personal innovativeness positively influences behavioral intention to adopt digital wallets.

#### **H4b**

Users’ behavioral intention to adopt digital wallets mediates the relationship between perceived personal innovativeness and continued usage behavior of digital wallets.

### Perceived social influence

With the intensity of social networks being practiced daily, potential users of new technology are heavily influenced to use digital wallets. Previous research finds that the degree to which potential users believe their social network services affect the intention to use mobile payment and its actual use [18, 63, 81]. Social Influence is majorly focused on how the technology acceptance process is focused in greater detail, depending on the attributes of people around the users [81]. Perceived social influence is the primary driver of behavioral intention, followed by performance expectancy and personal attitude, while trust did not affect the customers' behavioral intention [15]. Social Influence allows the consumers to gather information from other users regarding their experience and the service users consider the information provided by peers on social media [29]. Social Influence is the strongest predictor of behavioral intention [15].

#### **H5a**

Perceived social influence positively influences behavioral intention to adopt digital wallets.

#### **H5b**

Users’ behavioral intention to adopt digital wallets mediates the relationship between perceived social influence and continued usage behavior of digital wallets.

### Perceived insecurity

To fully adopt technology like digital wallets, there have always been barriers like perceived insecurity due to privacy issues [67]. The digital wallet service provider directly impacts consumer intentions to use m-payment services. In contrast, a lack of consumer trust may impede the uptake of this type of payment service [9]. In Iran, a study on the factors that affect trust in online banking was discovered, and their influences were managed, which provided support concerning digital wallets [56]. A recent study shows current users are highly concerned about the issue of trust with entities involved in the digital wallet payment process and activities as they are very much aware of giving the digital wallet service providers their personal information (e.g., telephone number, date of birth, address, credit card number) when conducting such payment transactions [9]. While addressing digital wallets, in general, a consumer uses four personality traits: optimism, innovativeness, discomfort, and insecurity. Optimism and innovativeness are drivers of TR, while discomfort and insecurity are inhibitors [7]. Insecurity of the consumers can be reduced by providing a positive user experience [101]. Trust and mutual complementarity are also proven to promote behavioral intention, which can increase digital wallet usage [67]. This willingness to have confidence and reliance on an exchange partner is a cornerstone in building trust and helps customers make the behavioral intention of labeling an exchange partner trustworthy [23, 88].

#### **H6a**

Perceived insecurity positively influences behavioral intention to adopt digital wallets.

#### **H6b**

Users’ behavioral intention to adopt digital wallets mediates the relationship between perceived insecurity and continued usage behavior of digital wallets.

### Relationship between behavioral intention and continued usage of digital wallets

The literature suggests that users' behavioral intention refers to the willingness to perform a particular behavior and behavioral intention is the antecedent of usage behavior [2]. The past study findings indicated a positive and significant association between behavioral intention and continued usage behavior in information technology [85–87, 102]. In internet banking adoption, the researchers also found similar findings that behavior intention has a positive and influential impact on continued usage behavior [54, 65]

#### **H7**

Users' behavioral intention to adopt digital wallets is positively associated with continued usage behavior.

### The moderating role of perceived technological innovativeness

Technological innovativeness is "the perceived degree of newness and improvement over existing alternatives" [50]. To adopt an innovation, a person must perceive the idea, behavior, or product as innovative [7]. Motivation aspects of the invention, such as social status and cost, are considered elements of relative advantage. In this case, the e-wallet is a method of redefining the conventional payment method used by the consumer because of the low cost and fulfilling the social status [76]. Generally, firms that showcase their technological superiority in their advertisements can attract more customers, leading to a better return on their innovation-driven ventures [69]. Research also showed that companies' customers with new superior market technology had a better online engagement and were more eager to advocate the service providers' technology and benefits [63]. Customers’ technology familiarity is one of the main factors to enhance the perception of useful platform innovativeness [77].

#### **H8**

Perceived technological innovativeness significantly moderates the relationship between behavioral intention to adopt digital wallets and continued usage.

### Control variables

**Fig. 1:**
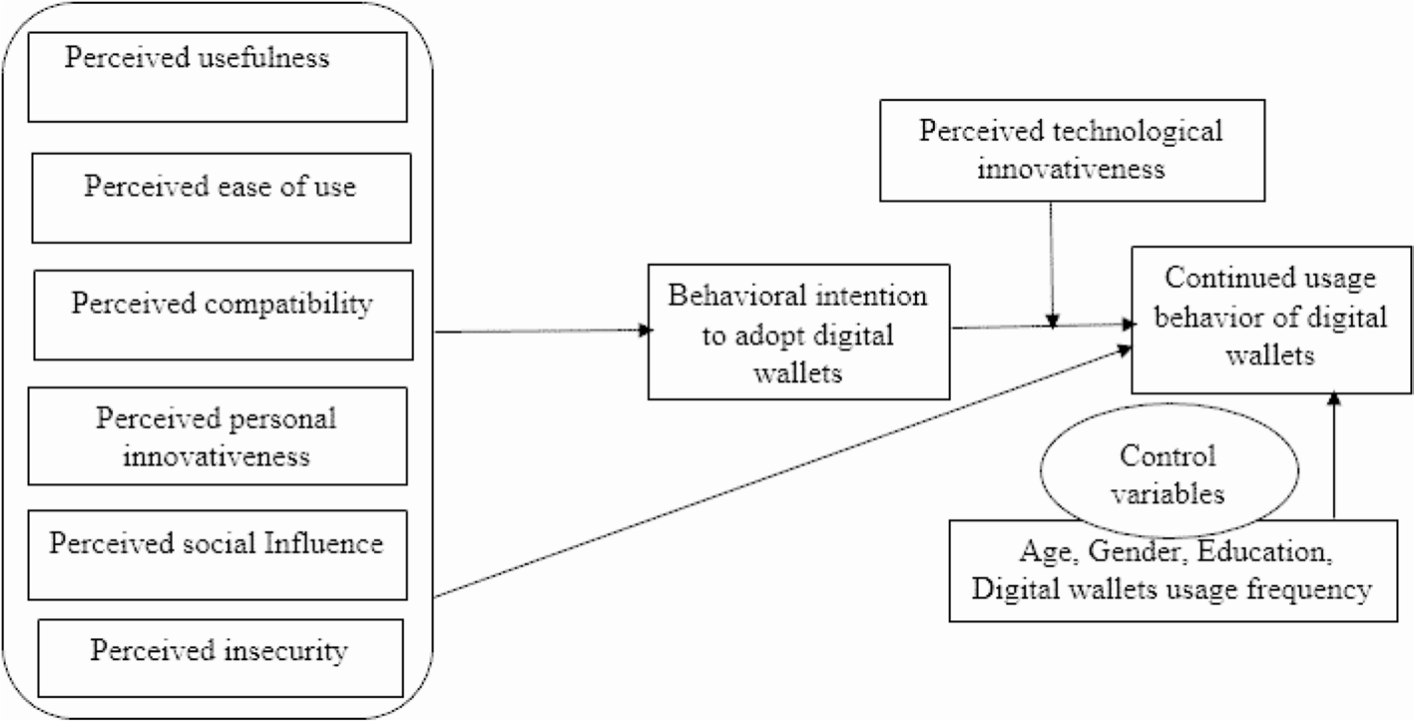
A proposed research model

Many types of research depicted how personal factors, such as age, gender, and experience, were included as moderating constructs or control variables [9, 49, 75]. Previous research showed that the more interaction between provider and customer, the higher the customer satisfaction and loyalty [9]. Schmidthuber et al. [76] showed no change in the significance level of the controls except for income, and even there, the difference is insignificant. A few types of research showed that access to banking services remains limited in rural areas and for lower-income populations, which is a significant constraint in accessing primary data from user behavior [77]. Some literature shows that males commonly associate socio-demographic characteristics with a higher interest in digital wallets [18]. People with higher education and higher income have higher behavioral intentions toward digital payment systems [15] (Fig. 1).

## Research methodology

The current study investigates the behavioral intention to adopt digital wallets of Bangladeshi consumers' perspectives and the continued usage of digital wallets in payment services technologies.

### Research context

The technological adoption in everyday life of the people of Bangladesh has contributed to enormous progress in digital payment services that help to contribute to the cashless economy [34]. People are more willing than in previous years to undertake digital payment services, including mobile payment, synonymously addressed as digital wallets. The COVID-19 pandemic has compelled people, especially the underprivileged, to adopt digital wallets to enhance the Bangladesh government’s safety net programs [34]. The study was focused on Bangladesh perspectives which now has a 60% mobile internet penetration rate [72], and the rate of accepting mobile payment services has increased by 7.7% [34]. Digital payment services are getting popular among diverse socio-demographic groups to make their usage more restful. Bangladesh's banks and financial instructions provide digital payment services to customers [62]. According to recent data age group 15–24 years in Bangladesh, the literacy rate was about 94.46% among the residents [82]. The current facts justified testing our proposed research problem in the Bangladesh context.

### Sampling and data collection

To analyze the data for this study, the researcher collected the required primary data through a structured quantitative questionnaire survey form. The research questionnaire was created in English, focusing on this study's target respondents. The researchers sent a consent form including the survey questionnaire to know the respondents’ will to participate in this survey. The researchers also provided the necessary information for this survey, including the digital wallets’ basic information and the user experiences of balance checking, transforming money, and conducting payments via mobile devices [23]. The participants were not given any financial benefits in participating in this online questionnaire survey. The researchers used nonprobability purposive sampling to analyze the hypothesized relationships [13]. A web-based survey using a list-based sampling frame was used in this study; the specified target users of digital wallets and the universities listed the respondents' e-mails collected to send the invitation link [22]. Besides, the researchers used personal contacts to post the survey links on multiple social media platforms such as Facebook and Instagram. A similar medium of data collection tools was previously used [13]. To address the target users, the research questionnaires were distributed through e-mail, Google classroom, and social media platforms such as Facebook and Instagram from January to February 2022 [7].

Using online surveys has several advantages: making the responses faster and greater accurate, lowering the cost of collecting data, and less effort to administer [22]. The questionnaire was divided into two sections. The first section included the socio-demographic profile of the respondents, and the second section addressed the model constructs of the independent study constructs, dependent construct, and moderating construct. The respondent’s age group of 18 was considered and had experience in digital wallets with mobile payment services in Bangladesh. About one-thousand (1000) generalized people from students and alumni from universities in Dhaka, Bangladesh, were communicated by an e-mail questionnaire survey and Facebook messenger invitation. A hyperlink to the online poll was encompassed in the e-mail to get the response faster [7, 63]. The researchers targeted 500 respondents from different regions of Dhaka city, Bangladesh, who currently use digital wallets for payment purposes. A set of 350 completed questionnaires was returned, and twenty questionnaires were removed due to respondents' errors in filling out the survey form. A total of 330 responses were found usable that finalized to conduct the data analysis, and the response rate of 66% indicated adequate survey research. Initially, the respondents' details showed that most of the respondents' age was 18 to below 35 years and revealed the respondents' consistency from the previous research [7].

The researchers scrutinized all the responses to ensure the data accuracy and validity of the survey results. They laid off the too many missing data values, same answers given for all questions, incomplete questionnaire form, or who had no digital wallets payment services experiences [23]. The sample size of this study is sufficient to get the reliable SmartPLS3.0 results which meet the generally accepted '10 times rule of thumb that indicates the minimum sample size as ten times the most complex relationships in the research model [12]. This model's behavioral intention to adopt digital wallets has eight constructs; the required respondent sample size would be 80. The researchers also applied the non-response bias test, which did not appear as a significant concern, and we could not find the essential differences between the respondents who filled the questionnaire form early and those who responded late concerning crucial measures in the 5% significant level [23].

### Measurement scales development

For research purposes, the researchers developed a self-reporting questionnaire comprising 28 items designed using the continued usage intention of digital wallets in Bangladesh perspectives. The survey items were adopted from related literature on UTAUT2 Venkatesh et al. [87], DOI [71], and TRI2.0 [64]. This study measures dependent, moderating, and all independent constructs using a 5-point Likert scale from strongly disagree (1) to agree (5) strongly. Perceived usefulness and perceived ease of use were adopted from Venkatesh et al. [87]. Both of the constructs were measured using a 4-item scale. The perceived compatibility construct was measured using a 3-item scale adapted from Parasuraman and Colby [64]. The perceived personal innovativeness construct was measured using a 3-item scale and adopted from Rogers [71] and Parasuraman and Colby [64], and perceived social influence was adopted from Parasuraman and Colby [64] and Venkatesh et al. [87], whereas used 3-item scale from the existing literature. Besides, Perceived insecurity used a 3-item scale adapted from [64]. The behavioral intention was adopted from Venkatesh et al. [87] and measured using a 3-item scale. Continued usage intention was measured using a 2-item scale adapted from Zhou [101]. Finally, the moderating construct perceived technological innovativeness was measured using a 3-item scale adapted from Parasuraman and Colby [64]. Table 1 shows the details of measurement scales and the statement overview.

**Table 1:** Measurement items

Construct	Items	Statements	Sources
Perceived usefulness	PU	PU1: Using digital wallets improve the course of my daily life PU2: Using digital wallets in my everyday life increases my productivity PU3: Using digital wallets enhances the effectiveness of my daily life PU4: Using digital wallets would help to manage my expense better	Venkatesh et al. [87]
Perceived ease of use	PEU	PEU1: The payment process with mobile apps is clear and understandable PEU2: The payment process with mobile apps does not require a lot of mental effort PEU3: I find it convenient to pay digitally PEU4: It is easy to follow all the steps to using the mobile payment system	Venkatesh et al. [87]
Perceived compatibility	PC	PC1: Using digital wallets fit well with my lifestyle PC2: Using digital wallets does well with the way I like to purchase products and services PC3: I would appreciate using digital wallets instead of traditional modes of payment	Parasuraman and Colby [64]
Perceived personal innovativeness	PPI	PPI1: I would look for ways to experiment with new information technologies PPI2: Among my friends/ colleagues, I am usually the first to try out new information technologies PPI3: In general, I do not mind trying digital payment application that is new in the market	Parasuraman and Colby [64], Rogers [71]
Perceived social influence	PSI	PSI1: People who are important to me think that I should use digital wallets PSI2: People who influence my behavior think that I should use digital wallets PSI3: People whose opinions I value prefer that I should use digital wallets	Parasuraman and Colby [64], Venkatesh et al. [87]
Perceived insecurity	PI	PI1: I just don’t trust any digital payment mechanism PI2: I feel insecure in using digital payment services PI3: I am concerned about my online privacy	Parasuraman and Colby [64]
Behavioral intention	BI	BI1: I am likely to use digital wallets soon BI2: I am open to using digital wallets when the opportunity arises BI3: I intend to use digital wallets when the opportunity arises	Venkatesh et al. [87]
Continued usage intention	CUI	CUI1: I will frequently use the digital wallets platform CUI2: I will continue to use my digital wallets apps	Zhou [101]
Perceived technological innovativeness	PTI	PTI1: Digital wallets are technologically new and innovative PTI2: The technology of the digital wallets platform allows me to receive the best service PTI3: I am confident that digital wallets will be at the forefront of future payment platforms	Parasuraman and Colby [64]

### Statistical tool

The researchers analyzed the data using IBM Statistical Package for Social Sciences (SPSS) version 25 to analyze the sample of respondents, using mean and frequency. Besides, to verify the proposed theoretical model of this study and identify the significant constructs and the mediating and moderation effects of behavioral intention to adopt digital wallets and the technological innovativeness, a Partial Least Structural Equation Modelling (PLS-SEM) was performed using SmartPLS 3.0. They considered the study sample size of 330 and the Partial Least Square (PLS) through a multivariate technique applied to test structural models [7].

### Common method bias (CMB)

To ensure the reliability and validity of the proposed model, the researchers preliminary investigated the Common method bias (CMB). The Variance Inflation Factor (VIF) was analyzed, and the previous study findings indicated that VIFs values below 3.3 are considered the threshold level [42]. Besides, the acceptance of a VIFs value of 5.0 was indicted as the maximum level [31]; furthermore, Hair Jr et al. [27] considered 10.0 as the maximum level of VIFs values. Considering the previous findings, the present study results of VIFs showed no collinearity issue, and the model was unaffected by any common method bias (CMB) issue.

## Data analysis and results

### Demographic characteristics

Table 2 shows the demographic characteristics of the 330 respondents. From the mentioned information, it can be assessed that males surpassed females (192 vs. 138), accounting for 58.2%. Most respondents were between 22 and 25 years old, accumulating 45.2% of the total responses. The respondents were university students in their undergrad education level, and the majority, 30% of the respondents, have had a Master’s degree. Considering Table 2, weekly, 0–3 times the respondents used digital wallets, 65.8%. bKash appeared to be one of the favored digital wallet payment services among the respondents, 97%, followed by Nagad 35.2% and Nexus Pay 24.8%. bKash is the first e-wallet service in Bangladesh, introduced in 2010; rated one of the finest and adored digital payment services based on its functionality and immense acceptance [34].

**Table 2:** Demographic profile of the respondents

Demographic attribute	Category	Frequency	Percentage (%)
Gender	Male	192	58.2
	Female	138	41.8
Age	18–21	81	24.5
	22–25	149	45.2
	26–30	87	26.4
	31–35	12	3.6
	Above 35	1	0.3
Education	Undergrad 1st Year	50	15.16
	Undergrad 2nd Year	42	12.73
	Undergrad 3rd Year	51	15.45
	Undergrad 4th Year	87	26.36
	Masters	100	30.30
Frequency of digital wallets payment services usage (in a week)	0–3 times	217	65.8
	4–8 times	76	23
	More than eight times	37	11.2
Frequently used digital wallets payments (multiple options)	bKash	320	97
	Nagad	116	35.2
	Upay	12	3.6
	Trust Axiata Pay (tap)	22	6.7
	Rocket	82	24.8
	Nexus Pay	36	10.9
	Cellini	11	3.3
	Citytouch	9	2.7
	Others	14	4.2

*Source*: Researcher’s computation

### The measurement model assessment

In this study, reliability and validity assessments were analyzed using SmartPLS3.0. Items factor loadings were assessed based on a loading threshold of 0.6, Cronbach's alpha (*α*), Composite Reliability (CR), and Average Variance Extracted (AVE) were also examined, and the cut-off values were 0.7, 0.7, and 0.5, respectively. Eventually, the Fornell–Larcker criterion model examined the squared root of AVE to measure the potentially overlapping constructs that were used to ensure discriminant validity [21, 26]. Table 3 showed that all the items lower than 0.6 were removed, and thus two of them from perceived insecurity (PI1, PI2) were excluded from further analysis. The calculated Cronbach’s alpha (*α*) values range from 0.728 to 1.0, which indicated more significance than 0.7 and demonstrated the high reliability of the data [61]. Correspondingly, all the composite reliability (CR) values ranged from 0.839 to 1.0, higher than the recommended value of 0.7, and assured reliability [28]. In the end, the average variance extracted (AVE) of each construct examined more than 0.5. The study calculated results ranging from 0.637 to 1.0, which also demonstrated the validity of the factors [28].

**Table 3:** Construct reliability and the results of the outer model

Constructs	Measurement items	Loadings	*α*	CR	AVE	*R*^2^
Perceived Usefulness	PU1 PU2 PU3 PU4	0.874 0.868 0.913 0.642	0.845	0.898	0.691	
Perceived Ease of Use	PEU1 PEU2 PEU3 PEU4	0.881 0.844 0.881 0.909	0.902	0.932	0.773	
Perceived Compatibility	PC1 PC2 PC3	0.914 0.901 0.894	0.887	0.930	0.816	
Perceived Personal Innovativeness	PP1 PPI2 PPI3	0.887 0.778 0.721	0.728	0.839	0.637	
Perceived Social Influence	PSI1 PSI2 PSI3	0.918 0.944 0.925	0.921	0.950	0.863	
Perceived Insecurity	PI3	1.000	1.000	1.000	1.000	
Behavioral Intention	BI1 BI2 BI3	0.924 0.936 0.918	0.917	0.947	0.857	0.629
Continued Usage Intention	CUI1 CUI2	0.948 0.954	0.894	0.950	0.904	0.768
Perceived Technological Innovativeness	PTI1 PTI2 PTI3	0.901 0.886 0.907	0.880	0.926	0.807	

*Source*: SmartPLS3.0 analysis

### Discriminant validity

The researchers examined the discriminant validity by evaluating the values of the outer and inner variance inflation factor (VIF). The discriminant validity can be measured in the Fornell–Larcker criterion model and cross-loadings [30]. According to the study’s evaluated results, the highest outer VIF value was 4.036, while the highest inner VIF value was 7.326, lower than the cut-off value of 10.0. The data have had no multicollinearity issue [27]. The following Table 4 demonstrates the Fornell and Larcker. We observed the correlation of all latent constructs and compared them with the square root of their respective average variance-extracted values in the correlation [61]. Table 4 also observes that AVE's square root (in italic) is higher than the correlation values of other constructs on both horizontal and vertical sides, making it evident that there were no discriminant validity issues.

**Table 4:** Discriminant validity-Fornell and Larcker criterion model

Constructs	BI	CUI	PC	PEU	PI	PPI	PSI	PTI	PU
Behavioral intention (BI)	*0.926*								
Continued Usage Intention (CUI)	0.787	*0.951*							
Perceived Compatibility (PC)	0.694	0.781	*0.903*						
Perceived Ease of Use (PEU)	0.726	0.785	0.801	*0.879*					
Perceived Insecurity (PI)	0.394	0.316	0.283	0.312	*1.000*				
Perceived Personal Innovativeness (PPI)	0.602	0.663	0.712	0.676	0.266	*0.798*			
Perceived Social Influence (PSI)	0.485	0.547	0.610	0.536	0.321	0.630	*0.929*		
Perceived Technological Innovativeness (PTI)	0.778	0.853	0.756	0.770	0.412	0.652	0.596	*0.898*	
Perceived Usefulness (PU)	0.721	0.777	0.751	0.772	0.309	0.632	0.610	0.768	*0.831*

Italic values represent the square root of AVE

Besides, in Table 5, the researchers showed the Heterotrait-Monotrait Ratio (HTMT) analysis. The recommended HTMT is below 0.9 [24], and the study results passed the rule of thumbs of the recommended value. The presented results suggested no multicollinearity issue in further analysis [28, 30].

**Table 5:** Heterotrait-Monotrait Ratio (HTMT)

Constructs	BI	CUI	PC	PEU	PI	PPI	PSI	PTI	PU
Behavioral intention (BI)									
Continued usage intention (CUI)	0.869								
Perceived compatibility (PC)	0.769	0.875							
Perceived ease of use (PEU)	0.797	0.871	0.892						
Perceived insecurity (PI)	0.411	0.333	0.300	0.328					
Perceived personal innovativeness (PPI)	0.685	0.777	0.850	0.782	0.279				
Perceived social influence (PSI)	0.527	0.602	0.674	0.586	0.332	0.779			
Perceived technological innovativeness (PTI)	0.865	0.961	0.855	0.862	0.438	0.771	0.664		
Perceived usefulness (PU)	0.815	0.887	0.862	0.883	0.336	0.776	0.700	0.886	

## The structural model assessment

### Structural model analysis

To assess the proposed hypotheses, initially, the regression analysis was applied. In Table 3, the regression analysis findings have been inserted using SmartPLS3.0. *R*^2^ values indicated that behavioral intention to adopt digital wallets and continued usage intention had *R*^2^ values of 0.629 (62.9%) and 0.768 (76.8%), respectively. The *R* square values demonstrated the good interpretive strength of the dependent constructs.

### Test of hypotheses

The PLS-SEM output has been presented to evaluate the statistical significance of the proposed theoretical model, followed by the proposed hypotheses outcomes in Table 6. The path analysis direct results of perceived usefulness, ease of use, compatibility, personal innovativeness, social influence, insecurity, behavioral intention, and technological innovativeness are illustrated in the following Table 6. Hypothesis H1a, perceived usefulness significantly influences behavioral intention to adopt digital wallets (*β* = 0.320, *t* = 5.524, *p* = 0.000), supports hypothesis H1a. Hypothesis H2a, perceived ease of use substantially impacts users' behavioral intention to adopt digital wallets (*β* = 0.262, *t* = 2.937, *p* = 0.003), supported H2a. The direct path of perceived compatibility has significantly impacted users' behavioral intention (*β* = 0.165, *t* = 2.054, *p* = 0.040) that supported H3a. Besides, perceived personal innovativeness is not statistically significant on users' behavioral intention to adopt digital wallets (*β* = 0.106, *t* = 1.831, *p* = 0.068), which rejected hypothesis H4a. Consequently, the noted fact that perceived social influence also was not statistically significant and had a negative effect on the proposed hypothesis H5a, and the results showed as (*β* =  − 0.069, *t* = 1.247, *p* = 0.213). Moreover, perceived insecurity has significantly impacted users' behavioral intention (*β* = 0.161, *t* = 4.075, *p* = 0.000), supporting the proposed hypothesis H6a. Lastly, users' behavioral intention to adopt digital wallets significantly influences continued usage intention (*β* = 0.309, *t* = 5.817, *p* = 0.000), supporting hypothesis H7. The moderation effect of perceived technological innovativeness has not considerably impacted behavioral intention and continued usage intention among the users (*β* =  − 0.020, *t* = 1.032, *p* = 0.302), resulting in the rejection of hypothesis H8. The respondents’ age, gender, education, and digital wallet usage frequency were control variables with no significant effect. In summary, we stated that H1a, H2a, H3a, H6a, and H7 supported the proposed hypotheses; on the contrary, hypotheses H4a, H5a, and H8 were rejected based on the outcome results evaluation. Furthermore, in Table 6, the researchers also explained the f square and the effect size of the endogenous variables [14]. H1a found a medium effect size among the proposed hypotheses, and H8 found a large effect size; hypotheses H2a–H7 demonstrated a small effect size.

**Table 6:** Hypotheses testing

Hypothesis	Structural paths	Path coefficients (*β*)	*T* value	*P* value	*f* square	Effect size	Decision
H1a	PU > BI	0.320	5.524	0.000	0.154	Medium	Supported
	PU > CUI	0.099	3.791	0.000			
H2a	PEU > BI	0.262	2.937	0.003	0.019	Small	Supported
	PEU > CUI	0.081	2.634	0.009			
H3a	PC > BI	0.165	2.054	0.040	0.005	Small	Supported
	PC > CUI	0.051	1.877	0.061			
H4a	PPI > BI	0.106	1.831	0.068	0.021	Small	Not supported
	PPI > CUI	0.033	1.661	0.097			
H5a	PSI > BI	− 0.069	1.247	0.213	0.037	Small	Not supported
	PSI > CUI	− 0.021	1.224	0.222			
H6a	PI > BI	0.161	4.075	0.000	0.082	Small	Supported
	PI > CUI	0.050	4.170	0.000			
H7	BI > CUI	0.309	5.817	0.000	0.081	Small	Supported
H8	PTI*BI > CUI	− 0.020	1.032	0.302	2.492	Large	Not supported

### Testing mediated effects

In Table 7, the behavioral intention is presented as the mediation of this following study, and the results are shown in the next section. The hypothesis H1a and H2a were statistically significant and supported the hypotheses in the direct path. Yet, indirect effects of significance level in the mediation testing; the behavioral intention mediates the relationships of perceived usefulness, perceived ease of use, and perceived insecurity. This also supported hypotheses H1b, H2b, and H6b of this study. Besides, the specific indirect effects of the significant mediation relationship testing among the hypotheses of perceived compatibility perceived personal innovativeness, and perceived social influence rejected the proposed hypotheses H3b, H4b, and H5b.

**Table 7:** Significance of specific indirect effects

Hypothesis	Indirect path	Ptah coefficients (*β*)	*T* value	*P* value	Decision
H1b	PU > BI > CUI	0.320	3.791	0.000	Supported
H2b	PEU > BI > CUI	0.262	2.634	0.009	Supported
H3b	PC > BI > CUI	0.165	1.877	0.061	Not supported
H4b	PPI > BI > CUI	0.106	1.661	0.097	Not supported
H5b	PSI > BI > CUI	− 0.069	1.224	0.222	Not supported
H6b	PI > BI > CUI	0.161	4.170	0.000	Supported

*p* < 0.05, *p* < 0.001

Table 8 presents the results of the degree of mediation through variance accounted for (VAF). The results suggested that users’ behavioral intention to adopt digital wallets partially mediate the proposed hypothesis relationships of perceived usefulness (H1b), perceived ease of use (H2b), perceived personal innovativeness (H4b), perceived social Influence (H5b), and perceived insecurity (H6b) in between continued usage intention to adopt the digital wallets facilities. In consequence, the hypothesized results were supported. Besides, users' behavioral intention to adopt digital wallets did not find any mediation between perceived compatibility and continued usage intention; on the contrary, the proposed hypothesis rejected the mediation effect.

**Table 8:** Degree of mediation through Variance Accounted For (VAF)

Hypothesis	Mediated paths	Indirect path *I* = (*a* * *b* * *c*)	Direct path (*D* = *T* − *I*)	Total effect (*T*)	VAN (*I*/*T*) (%)	Results
H1b	PU > BI ˃ CUI	0.09984	0.1	0.19984	49.95	Partial mediation
H2b	PEU > BI > CUI	0.08174	0.082	0.16374	49.92	Partial mediation
H3b	PC ˃ BI ˃ CUI	0.05148	0.052	0.312	16.5	No mediation
H4b	PPI ˃ BI ˃ CUI	0.03307	0.033	0.06607	50.54	Partial mediation
H5b	PSI ˃ BI ˃ CUI	− 0.0215	− 0.022	0.0435	49.46	Partial mediation
H6b	PI ˃ BI ˃ CUI	0.05023	0.05	0.10023	50.12	Partial mediation

### Model fitness

The estimated value of goodness of fit (GOF) is 0.756, which indicates a good model fit, as shown in Table 9. Besides, the SRMR value of 0.063 showed an excellent model of fitness [28].

**Table 9:** Goodness of fit (GOF)

Constructs	AVE	*R*^2^
Perceived usefulness	0.691	
Perceived ease of use	0.773	
Perceived compatibility	0.816	
Perceived personal innovativeness	0.637	
Perceived social influence	0.863	
Perceived insecurity	1.000	
Behavioral intention	0.857	0.629
Continued usage intention	0.904	0.768
Average scores	0.818	0.699
AVE * *R*^2^	0.572	
GOF = √(AVE * *R*^2)^	0.756	

## Discussion

The present study aims to provide some provoking ideas about users’ behavioral intention to adopt digital wallets evaluating in collaboration with the technology acceptance model (TAM), Diffusion of Innovation (DoI), and Technology Readiness Index 2.0 (TRI 2.0). The mediating role of behavioral intention has been examined, besides the perceived technological innovativeness is also assessed in this theoretical model. The researchers tried to draw a technology literature review. To determine the technological-specific characteristics, they identified the constructs of perceived usefulness, ease of use, compatibility, personal innovativeness, social influence, and insecurity. The study outcomes demonstrated the direct and indirect effects on users' behavioral intention to adopt digital wallets at their convenience. The study findings showed that perceived usefulness significantly influenced behavioral intention to adopt digital wallets and supported hypotheses H1a and H1b. The previous literature also supported the current results in the context of Malaysia [7], South Korea [39, 40], and India [81].

Consequently, perceived ease of use had the direct and indirect pathways of positively significant relationships with users' behavioral intention. The proposed hypotheses H2a and H2b supported the study outcomes that had similarities with recent literature findings [7, 81]. Users’ perceived compatibility directly and significantly positively influenced behavioral intention to adopt digital wallets that supported the H3a, and our study findings are consistent with the following conclusions [76]. In Germany, Liébana-Cabanillas et al. [48] found an inconsistent and non-significant relationship between perceived compatibility and users' behavioral intention. On the contrary, the indirect, specific effects of digital wallets' perceived compatibility and continued usage (H3b) were statistically insignificant. The findings revealed that individual perceptions of technology, needs, and experiences affect every trait that differs across European and Asian territories [76]. In this study context, perceived personal innovativeness did not support the direct and indirect specific pathways of the proposed hypotheses H4a and H4b. The study evidence indicated the inconsistency with the previous literature of the past studies, whereas the perceived personal innovativeness was statistically significant and consistent with prior findings [20, 76, 93]. The construct perceived social influence was not statistically significant in direct and indirect pathways of coefficients, which rejected the proposed hypotheses H5a and H5b.

Nevertheless, in the Austrian context, the researchers found a significantly positive association between social influence and the individuals' way of adopting digital wallets [76]. Additionally, in European countries' perspectives, similar results showed the following social influence and behavioral intention to embrace digital wallet services [43, 46]. The perspectives of developing and developed countries on this construct might have significant influencing motivators to adopt digital wallets. In Bangladesh, people, especially the younger generation, who are more tech-savvy and prompt to learn new ideas, are now conveniently connecting with digital services. Users' perceived insecurity significantly influenced both direct and indirect pathways; as a result, the proposed hypotheses H6a and H6b were accepted. This study's findings indicated that the prior studies' results reveal a significant concern while using digital wallets [23, 43, 73, 97].

Users' behavioral intention significantly influenced the continued usage intention to adopt digital wallets, which supported the current study's proposed hypothesis H7. Previous literature suggested that perceived insecurity negatively affected behavioral and continued intention to adopt digital wallets daily [76]. As a result, users who think mobile payment services are risky feel less unwilling to adopt new technology. Considering the respondents of this study, most of them are highly educated to operate digital wallet apps and have the efficient knowledge and skills to use them [7]. The perceived technological innovativeness (H8) was inconsistent with the continued usage of digital wallets, which did not support the proposed hypothesis of this study's findings.

### Implications of the study findings

The present study findings have indicated several avenues to add to the theoretical contribution in the existing literature. The authors attempted to investigate the antecedents that encourage users to adopt digital wallets. The researchers contributed to the literature on digital wallets, whereas users' behavioral intention to adopt digital wallets by incorporating behavioral intention antecedents and continued usage intention. The current study has included the behavioral intention of digital wallets adoption and behavioral factors such as perceived usefulness, perceived ease of use, perceived compatibility, perceived personal innovativeness, perceived social influence, and perceived insecurity affecting the continued usage intention of digital wallets that can be utilized by the researchers in other service industries such as online banking, online shopping, online food ordering system and so on. The research findings highlighted the significance of the theoretical framework for a better understanding of digital wallets' strategic importance, considering the cashless society's present circumstances. Secondly, the present study escalates the experience of investigating the behavioral intention to adopt digital wallets as the mediating role in digital wallets adoption, a new construct not even examined in developing countries like Bangladesh perspective. Furthermore, the study highlights the existing literature identifying perceived technological innovativeness as the moderating role in the relationships between behavioral intention to adopt digital wallets and continued usage intention of digital wallets because individuals’ perceived technological innovativeness continuously affects the choices.

The findings of the research have several real-life implications. From this analysis, it is apparent that several factors must be considered to build a cashless society. Factors identified in this research will surely help the service providers develop their service process further so that people are more inclined to use this. For example, security issues are considered a significant concern for people regarding digital wallets. Digital wallet service providers should work on this concern to make this transaction process safe. Users' behavioral intentions were also affected by the system's user interface. Care must be taken to make the interface as easy as possible for customers. It is essential to mention that having a positive attitude toward the system does not eventually lead to purchasing the service. That is why the service providers must work closely with the service receivers to generate ideas on making the service innovative and valuable for them to use continuously. At the same time, providers of digital wallets can also work with government agencies to reduce the security risk so that the service receivers do not shy away from taking the service.

The present study aims to provide some provoking ideas about users' behavioral intention to adopt digital wallets evaluating in collaboration with the technology acceptance model (TAM), Diffusion of Innovation (DoI), and Technology Readiness Index 2.0 (TRI 2.0). The mediating role of behavioral intention has been examined, besides the perceived technological innovativeness is also assessed in this theoretical model. We discussed various factors that favorably influence or prohibit people from using disruptive technology in their daily lives (e.g., perceived usefulness, compatibility, personal innovativeness, and social influence) (i.e., perceived risk). No other prior study was conducted that used an integrated framework of the technology acceptance model (TAM), Diffusion of Innovation (DoI), and Technology Readiness Index 2.0 (TRI 2.0) to analyze the factors affecting the continued use of digital wallets. Our findings show that Individuals' acceptance of technology is directly influenced by aspects of the technology, the environment, and the individual, regardless of any hurdles preventing them from embracing the technology.

## Conclusions

The current study examined the effect of perceived usefulness, ease of use, compatibility, personal innovativeness, social influence, and insecurity on users' behavioral intention to adopt digital wallets, further on users' continued usage behavior of digital wallets, specifically in the Bangladesh context. The researchers also investigated the role of perceived technological innovativeness as the mediator. As more and more people are getting familiar with this digital wallet system, researchers are gaining interest in understanding the behavior patterns displayed by the users of this sector [47, 98]. However, it's not yet been clear about the factors affecting the continuous adoption of m-commerce as mixed findings are reported in various researches [35, 98]. In the context of Bangladesh, very little is known about the continued usage intention of digital wallets. Our study tried to build a model to identify the underlying factors that play a part in creating behavioral loyalty toward adopting the technology. This study makes a theoretical contribution by combining Technology Acceptance Model (TAM) [16], Diffusion of Innovation (DOI) [71], and Technology Readiness Index 2.0 (TRI 2.0) together. Providing utility is not enough to develop repeat purchasing behavior among the users. Factors like innovativeness and risk aversion must also be taken into consideration. Users will not fall for the benefits if trust is lacking in the initial stage. Trust-building measures must be taken. The findings of this study can certainly help those managers working in this sector improve their service.

### Limitations and future research directions

The following study is not above certain limitations. The authors noted that assessing the study's findings and contributions is crucial despite its limitations. Like every other study, this study has several limitations. First, such restriction is that respondents are 18–35 and are mainly urban-based. To create a cashless society, it should include people from all lifestyles. Therefore, future studies should incorporate respondents from various social strata. Future studies could also administer focus group discussions and depth interviews to better understand the respondent's viewpoints. Our paper used self-administered surveys, leading to problems like social desirability and incorrect reporting [7]. Face-to-face encounters with respondents, particularly those with lower educational backgrounds, are thought to be more effective in data collecting than self-administered surveys, which do not allow for any clarification of any doubts. Meantime, focus group discussion enables the researcher to explore further the underlying motives of accepting and rejecting digital wallets. Another limitation is that respondents unfamiliar with using digital payment wallets might have had difficulties evaluating the benefits of the technology. It is noteworthy that these data are collected at a single point, whereas consumers' attitudes can change with time. The longitudinal study can be considered in future studies to accommodate better insights. Finally, qualitative data collection and analysis could be an excellent option to believe in the future. The qualitative analysis provides a better understanding of the phenomenon in technology adoption [87]. Using the mixed method by combining qualitative and quantitative approaches can facilitate understanding of human technology adoption behavior. Future studies can undoubtedly use this mixed method to uncover the factors influencing the adoption of digital wallets. Furthermore, the present research ignored the cultural factors due to time and funding restrictions; nevertheless, future studies may thus examine the cross-cultural variations in the behavioral intention of digital wallet adoption. Other moderating variables, such as customer citizenship behavior and self-control efficiency, can be discussed in future research.

## Data Availability

The datasets used during the current study are available from the corresponding author on reasonable request.

## Abbreviations

TAM: Technology acceptance model
DoI: Diffusion of innovation
TRI 2.0: Technology Readiness Index 2.0
PU: Perceived usefulness
PEU: Perceived ease of use
PC: Perceived compatibility
PSI: Perceived social influence
PPI: Perceived personal innovativeness
PI: Perceived insecurity
BI: Behavioral intention
CUI: Continued usage intention
PTI: Perceived technological innovativeness
